# Should the Number of Metastatic Pelvic Lymph Nodes Be Integrated into the 2018 Figo Staging Classification of Early Stage Cervical Cancer?

**DOI:** 10.3390/cancers12061552

**Published:** 2020-06-12

**Authors:** Luigi Pedone Anchora, Vittoria Carbone, Valerio Gallotta, Francesco Fanfani, Francesco Cosentino, Luigi Carlo Turco, Camilla Fedele, Nicolò Bizzarri, Giovanni Scambia, Gabriella Ferrandina

**Affiliations:** 1 Dipartimento per la salute della Donna e del Bambino e della Salute Pubblica, Fondazione Policlinico Universitario A. Gemelli, IRCCS, UOC Ginecologia Oncologica, 00167 Roma, Italy; luigi.pedoneanchora@policlinicogemelli.it (L.P.A.); valerio.gallotta@policlinicogemelli.it (V.G.); francesco.fanfani@policlinicogemelli.it (F.F.); camilla.fedele@guest.policlinicogemelli.it (C.F.); nicolo.bizzarri@policlinicogemelli.it (N.B.); giovanni.scambia@policlinicogemelli.it (G.S.); gabriella.ferrandina@policlinicogemelli.it (G.F.); 2 Istituto di Ginecologia e Ostetricia, Università Cattolica del Sacro Cuore, 00167 Roma, Italy; 3 Dipartimento di Oncologia, UOC Ginecologia Oncologica, Gemelli Molise, 86100 Campobasso, Italy; francesco.cosentino@gemellimolise.it (F.C.); luigicarlo.turco@materolbia.com (L.C.T.); 4 Brest Care Unit, Mater Olbia Hospital, 07026 Olbia, Italy

**Keywords:** cervical cancer, staging system, FIGO, IIIC, lymph node, number of lymph nodes, number of metastatic lymph nodes, cut-off

## Abstract

Introduction: Lymph node status has become part of the new staging system for cervical cancer (CC). It has been shown that patients staged as IIIC1 had heterogeneous prognoses and, in some cases, experienced better outcomes than patients with lower stages. We evaluated the impact of the number of metastatic pelvic lymph nodes (MPLNs) among patients with stage IIIC1 cervical cancer. Methods: Survival analyses were conducted in order to identify the best cut-off prognostic value relative to the number of MPLNs. Disease free survival (DFS) was considered the main outcome. Results: 541 patients were included in the study. Eighty-nine patients were of stage IIIC1. The best prognostic cut-off value of the number of MPLNs was 2. Patients with >2 MPLNs (*n* > 2 group) had worse DFS compared with those having <2 (N1-2 group) (5 yr DFS: 54.7% vs. 78.1%, *p* value = 0.006). Multivariate analyses demonstrated that the extent of MPLNs had little impact on DFS and that replacement of IIIC1 staging with N1-2 and *n* > 2 grouping provided a better, statistically significant model (*p* value = 0.006). Discussion: Using a cut-off value of 2, the number of MPLNs could better predict prognostic outcomes within stage IIIC1 cervical cancer and have potential implications for therapeutic decision-making in the treatment of patients with stage IIIC1 CC.

## 1. Introduction

Cervical cancer represents one of the most common malignancies in women. The mortality rate is approximately 50%, worldwide [1]. Lymph node status is a major prognostic factor. Therefore, assessment of lymph node involvement is considered a cornerstone of staging and therapeutic management, which is independent of the characteristics of the primary tumor [2,3].

Recently, the International Federation of Gynecology and Obstetrics (FIGO) has acknowledged the impact of lymph node involvement on the staging system of cervical cancer [4]. Thus, patients bearing metastatic pelvic lymph nodes (LNs, assessed pathologically or at imaging) are upgraded to stage IIIC1, and patients with metastatic aortic LNs, are upgraded to stage IIIC2.

Some authors, however, have revisited their series in light of the new FIGO staging system, engendering some doubts concerning its reliability. For example, patients with 2018 FIGO stage IB3 were noted to experience a better clinical outcome compared with those with stage IIA1 [5], while patients staged IIIC1 experienced more favorable clinical outcomes than patients staged IIIA-IIIB [6].

The proportion of metastatic pelvic LNs in early stage cervical cancer is around 10–25% [3,5,7,8,9]. The reported 5-year disease-free survival (DFS) rate is 70–80% [2,8,9], while the 5 yr overall survival (OS) rate is 65–80% [3,5,10]. It should be acknowledged, however, that patients with metastatic pelvic LNs present with different risk profiles largely due to additional features that include lymph node size, site, morphology, number and whether or not they are bilateral [11,12,13,14]. 

In this context, most attention has been focused on the extent and number of metastatic pelvic LNs in early stage cervical cancer [12,13,14,15]. Biases, however, including the retrospective design of the studies, the heterogeneity of adjuvant treatments, and the adoption of different definitions of this variable (such as the absolute number of metastatic pelvic LNs, or the ratio between the number of metastatic pelvic LNs and the number of removed pelvic LNs, or the log odds of metastatic pelvic LNs) [13,14,15,16,17,18] did not lead to integration of that information in the staging system. It has been suggested that the absolute number of positive LNs might provide a more reliable and predictive way of classifying lymph node status [14,19]. Such integration of the number of metastatic loco-regional LNs has been utilized for the staging system of other common malignancies, such as the Tumor-Nodes-Metastasis (TNM) classification, as well as in the American Joint Committee on Cancer (AJCC) staging systems [20,21,22].

This retrospective study was aimed at evaluating the prognostic role of the number of metastatic pelvic LNs in clinically defined, early stage cervical cancer. Univariate and multivariate analyses of parameters formally employing the current staging classification were carried out in FIGO stage IIIC1p cervical cancer patients. 

## 2. Materials and Methods

### 2.1. Study Design and Inclusion Criteria

After approval by the Institutional Review Board (IRB number CICOG-30-10-19/64), data were retrieved from the electronic database of the Gynecologic Oncology Unit of Fondazione Policlinico Universitario A. Gemelli, IRCCS, Rome. Patient demographics, as well as clinical, pathological and follow up data were collected retrospectively, and all women provided written informed consent regarding the collection and processing of data for scientific purposes. 

Inclusion criteria for the present study consisted of age >18 years, histologically confirmed cervical cancer, preoperative clinical early stage according to the 2009 FIGO classification, and surgery as primary treatment consisting of radical hysterectomy and bilateral pelvic lymphadenectomy performed by traditional open or minimally invasive surgery [23,24,25]. Aortic lymphadenectomy was performed at the discretion of the attending physician, and, in the case of pelvic lymph nodes, determined intra-operatively to be involved with metastatic disease, after frozen section analysis. 

Patients were excluded from the present study if they had received neoadjuvant therapy prior to surgery, had concomitant malignant diseases in addition to cervical cancer, or had incomplete pathology or follow-up data. Patients assessed to have FIGO stage IA1 and IA2 pathology were excluded due to the very low risk of the presence of lymph node metastasis.

In this study, an attempt was made to reclassify all patients according to the 2018 FIGO staging system, and to define the significance of the number of metastatic pelvic LNs within the stage IIIC1p.

In order to identify the best cut-off prognostic value relative to the number of metastatic pelvic LNs per patient, the 25th, 50th and 75th percentiles of pelvic LNs distribution was adopted, a priori. Disease free survival (DFS), and overall survival (OS) were considered as oncological outcomes.

### 2.2. Statistical Analysis

The χ2 test or Fisher’s exact test for proportion were used to analyze the distribution of clinical and pathological variables among the different groups. DFS was defined as the time elapsed between surgery and recurrence or date of the last follow up. OS was defined as the time elapsed between surgery and death due to cervical cancer, or date of the last follow up.

Medians and life tables were computed using the product limit estimates method according to Kaplan Meier [26], and the log-rank test was used to assess statistical significance [27]. The Cox regression model with stepwise variable selection [28] was employed to analyze the data relative to clinical and pathological characteristics as predictors of DFS in each group.

Statistical Package for Social Sciences software version 25.0 (IBM Corporation, Armonk, NY, USA) was adopted to carry out all statistical calculations. The *p* value < 0.05 was considered to be significant for all analyses.

## 3. Results

**Figure 1:**
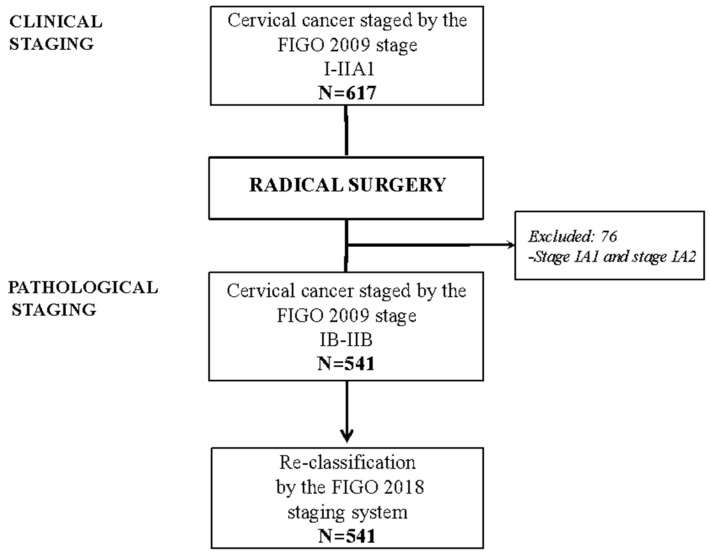
Flow chart of patients in the series. FIGO: International Federation of Gynecology and Obstetrics.

Between 1997 and 2018, 617 patients were clinically staged as FIGO 2009 I - IIA1, according to the inclusion criteria. After surgery, 76 patients classified as being stages IA1and IA2 were excluded, leaving 541 patients that were appropriate for statistical analysis (Figure 1).

The characteristics of the of the 541 patients included in this study are summarized in Table 1. Squamous cell histiotype was the most frequently observed neoplasm (*n* = 345, 63.8%). Few patients had a tumor size >4 cm (*n* = 50, 9.2%), disease in the upper third of the vagina (*n* = 59, 10.9%), or parametrial involvement (*n* = 42, 7.8%). Radical hysterectomy (Type B or C) was performed in over 90% of patients according to the Querleu—Morrow classification. Minimally invasive surgery accounted for >50% of procedures. Pelvic lymphadenectomy was performed in all cases, and the median number of pelvic LNs removed was 27 (range: 7–92). Aortic lymphadenectomy was performed in 116 patients (21.4%), and the median number of pelvic LNs removed was 11 (range: 2–38). 

Postoperatively, adjuvant treatment was administered to 292 patients (54.0%) according to their pathological findings, employing current guidelines. Table 2 shows the distribution of patients according to the 2009 and the 2018 FIGO staging systems. Of 418 patients staged as IB1, according to the FIGO stage 2009 system, 54 patients (12.9%) were reclassified as stage IIIC1p, and 7 patients (1.7%) as stage IIIC2p, respectively. Concerning stages IB2, IIA1 and IIA2 (FIGO 2009), the rates of reclassification to stage IIIC1p were 20.0%, 26.2% and 11.1%, respectively. Of significance, almost 40.5% of stage IIB patients were reclassified as stage IIIC1p, and 7.1% as stage IIIC2p.

Overall, of the 541 patients, 89 women (16.5%) were reclassified as stage IIIC1p. Thirteen patients (2.4%) with aortic LN metastasis were found to have concomitant metastatic pelvic LNs.

**Figure 2:**
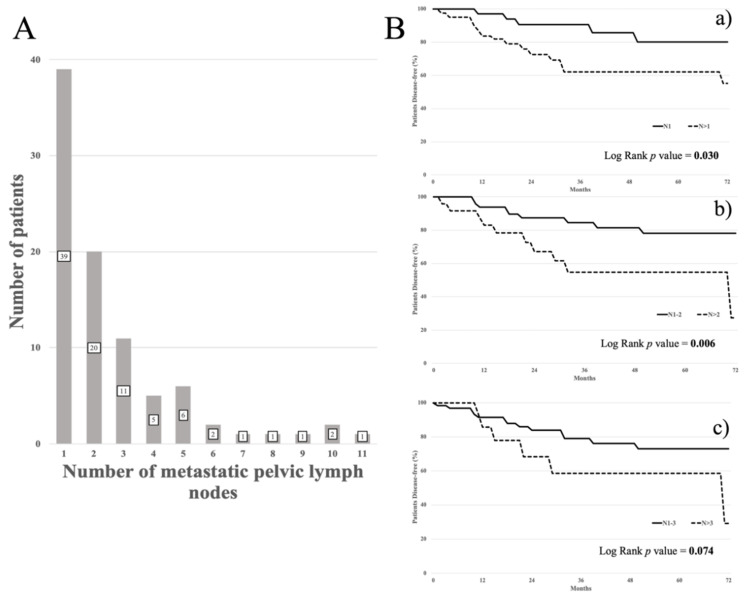
(**A**) Distribution of the number of metastatic pelvic lymph nodes per patient. (**B**) Disease free survival according to the cut off value of the number of metastatic pelvic lymph nodes representing the 25th (panel a), 50th (panel b), 75th (panel c) percentiles.

As for February, 2020, the median follow up of the entire cohort was 47 months (range: 3–302 months). There were 117 patients that had sustained a relapse of disease and 41 deaths. As shown in Figure 2A, the range of the number of metastatic pelvic LNs per patient was between 1 and 12. The 25th percentile represented 1 LN, the 50th percentile, 2 LNs, and the 75th percentile, 3 LNs.

As shown in Figure 2B, the best prognostic cut-off value for the number of metastatic pelvic LNs was represented by the median value. In patients with >2 metastatic pelvic LNs, the DFS was worse compared with patients harboring 1 or 2 metastatic pelvic LNs (5 yr DFS: 54.7% vs. 78.1%, *p* value= 0.006) (panel b). Patients with >1 positive pelvic LN showed a 5 yr DFS of 62.0% vs. 80.0% in patients with only 1 metastatic pelvic LN (*p* value = 0.030) (panel a). Patients with >3 pelvic LNs were shown not to differ in clinical outcome compared with cases in which there were 1 or 3 metastatic pelvic LNs (5 yr DFS: 58.4 % vs. 73.0%, *p* value = 0.074) (panel c).

Based on these results, patients with stage IIIC1p were divided into two subgroups: Those cases in which there were 1 or 2 metastatic pelvic LNs (N1-2), and those with metastatic disease in >2 pelvic LNs (*n* > 2). The distribution of pathological features according to the N1–2 and *n* > 2 groups (Table S1) was also analyzed. A tumor size > 4 cm and parametrial involvement were more frequently documented in the *n* > 2 group. The supplement to Table 2 shows the distribution of the pattern of recurrence according to status and number of metastatic pelvic LNs: Pelvic and lateral relapse(s) were reported more frequently in patients with negative pelvic LNs, while distant or mixed sites of disease were more prevalent in patients with metastatic pelvic LNs (*p* value = 0.0039).

**Figure 3:**
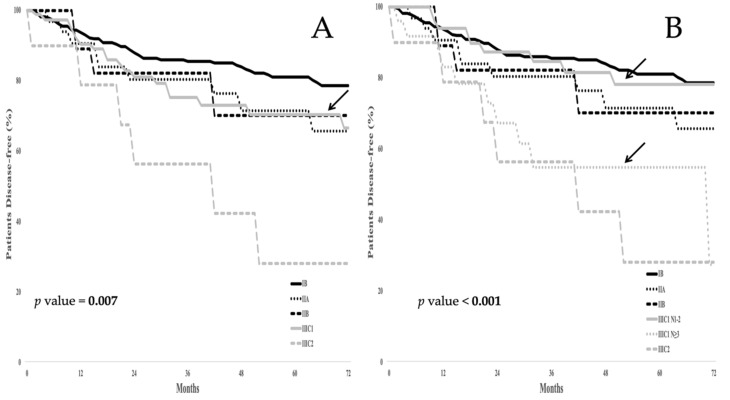
(**A**) Disease free survival (DFS) in the whole series according to the FIGO 2018 staging system; (**B**) DFS in the whole series according to the proposed FIGO 2018 staging system. Arrows highlight the DFS curve for stage IIIC1 in panel A, as well as the subgroups N1-2 and *n* > 2 in panel B.

Figure 3A shows the prognostic scenarios of all patients according to the standard 2018 FIGO staging system. The 5 yr DFS was 80.9% for stage IB, 71.5% for stage IIA, and 70.3% for stage IIB. Regarding stage IIIC1p, the 5 yr DFS was 70.3%, while the 5 yr DFS for patients staged IIIC2p was 28.1%.

Figure 3B shows the clinical outcome of all patients following the introduction of the N1–2 and *n* > 2 groups. Replacement of the system classifying patients as stage IIIC1p with one that classifies patients into groups as either N1–2 or *n* > 2 provides for a better determinate of prognosis since patients harboring >2 metastatic pelvic LNs experienced a 5 yr DFS of 54.7% compared with just 78.1% in patients with 1 or 2 metastatic pelvic LNs.

The supplement to Figure 1 demonstrates that the replacement of the system that stages patients as IIIC1p with one that assigns them to groups N1–2 or *n* > 2 (the number of LNs with metastatic disease) is a better determinant of overall survival. Patients assigned to the N1-2 group had a 5 yr OS of 84.7%, while those in the *n* > 2 group experienced a 5 yr OS of 55.3%.

No difference in the 5 yr DFS rate was observed between patients with negative pelvic LNs and those in the N1-2 group: The 5 yr DFS rate was 79.5% in negative pelvic LN patients vs. 78.1% in N1–2 group (*p* value = 0.642). Conversely, no statistical difference was observed between the *n* > 2 group and patients with metastatic aortic LNs: The 5 yr DFS was 54.7% in the *n* > 2 group vs. 28.1% in FIGO IIIC2p (*p* value = 0.718). In the reexamined multivariate analysis utilizing a system employing the number of pelvic LNs exhibiting metastatic disease, tumor size, as well as the extent of metastatic pelvic LNs remained statistically significant independent variables as they related to DFS (Table 3, *p* value = 0.006).

## 4. Discussion

In this report, data from a single institution are presented as a retrospective study, demonstrating that the number of pelvic LNs exhibiting metastases may be of importance when utilized in the FIGO 2018 staging system of cervical cancer patients.

The number of metastatic pelvic LNs was a better predictor of the prognostic outcome within stage IIIC1p patients. Specifically, the difference between the 5 yr DFS rate in cervical cancer patients with 1 or 2 metastatic pelvic LNs (78.1%) compared with those women having >2 metastatic pelvic LNs (54.7%) was approximately 23%, thus confirming the importance of the role that the extent of pelvic LN involvement may play in this disease, particularly at the selected cut off value of >2 metastatic pelvic LNs [5,10,11,29,30,31]. 

The prognostic value of utilizing the number of metastatic pelvic LNs was also confirmed in the OS analysis despite the fact that DFS might be related to several basic clinical, anatomical, and/or pathological features of the tumor, or by other factors, such as the site [32,33] and management of recurrence [34,35,36]. 

The increased number of metastatic LNs might also represent a marker of biological aggressiveness in terms of the potential for metastasis. Indeed, the pathogenesis of lymph node metastasis is complex, often including multiple mechanisms such as invasive potential, intravasation, extravasation and tissue-homing. Such mechanisms are promoted and sustained by different circulating cells and cytokines that are able to engender a micro-environment highly suitable for the promotion of lymph node tumor colonization [37]. Such a micro-environment may promote a more powerful clinical aggressiveness of tumors, of which the number of metastatic LNs could be useful as the marker. 

Nevertheless, these findings could be ascribed to the intrinsic features of local disease. Indeed, the frequencies of parametrial involvement and a primary tumor size >4 cm were higher in the *n* > 2 patient group compared with the N1–2 one, thus confirming previously reported data [6,10,13]. 

Yan et al. [38], investigating various ways of staging lymph node metastases according to the number and sites of LN involved among 155 patients, reported poor survival outcomes for women with 2 or more metastatic LNs. In the present study, a similar investigation was undertaken, but in the context of the other factors comprising FIGO staging. After adjusting for interaction between variables, tumor dimension and lymph node status, classification into groups defined as N0, N1–2, and *n* > 2 appeared to be the most important factor defining patient prognosis. As opposed to the TNM classification, in which T and N play independent roles [5,6], our model provided for a better stratification of patient prognosis, and also highlighted the independent role of primary, as well as loco-regional disease. A very recent, large scale study from the United States confirmed that worsened prognosis related to pelvic LN involvement largely varies with the extent of local tumor (T), suggesting that the influence of tumor, itself, might be as important, if not superior, to the presence of metastatic nodes (N) [31]. 

Interestingly, the 5 yr DFS of patients with only 1 or 2 metastatic pelvic LNs did not differ from patients with no pelvic LN involvement, as reported by others [14,39]. These findings should be confirmed in larger, well controlled prospective trials. Such information may have important implications on therapeutic decision-making for patients with only 1 or 2 metastatic pelvic LNs. Such patients, particularly in elderly women, or in those who are not candidates for chemo-radiotherapy, could be spared unnecessary morbidity. 

The presence of >2 metastatic pelvic LNs, however, denotes a completely different clinical setting, in which the 5 yr DFS rate is around 50%. This DFS rate is similar to that of stage IIIC2p patients. The National Comprehensive Cancer Network (NCCN) guidelines recommend pelvic chemo-radiation + vaginal brachytherapy (VBT) for adjuvant treatment of stage IIIC1p patients, and extended-field chemo-radiotherapy + VBT in patients with metastatic aortic LNs (https://www.nccn.org). However, considering the high risk of distant relapse(s), the role of adjuvant chemotherapy has recently been investigated in IIIC2p, as well as in IIIC1p cervical cancer patients [40,41,42]. In these studies, the use of systemic treatment versus chemo-radiotherapy in cervical cancer patients with high risk features appeared equivalent [36]. Thus, patients with metastatic pelvic LNs are likely to experience a higher rate of distant or mixed relapse(s) and dismal clinical outcomes, and, therefore, might be considered as candidates for the use of adjuvant chemotherapy.

Additionally, recent lines of evidence have shown that adoption of systemic chemotherapy after radiation treatment could act as consolidation treatment, potentially improving survival outcome in this patient population [43,44,45]. In this context, two phase III randomized studies are underway, investigating the postoperative role of chemo-radiotherapy, with or without additional chemotherapy in high risk early stage cervical cancer patients (the RTOG0724 trial, NCT00980954, and the Chinese study NCT03468010) (www.clinicaltrials.gov). 

This present study has several limitations, such as its retrospective design, the relatively small sample size, and the time frame involved, which could have limited the homogeneity of adjuvant treatment(s). An extension of this study is planned to include the MITO (Multicenter Italian Trials in Ovarian cancer) surgery group (www.mito-group.it) with additional collection of data relative to the issue of ovarian cancer treatment, providing further validation within Italian centers. 

## 5. Conclusions

Quantification of the number of lymph nodes exhibiting metastatic disease in women with ovarian cancer, specifically employing a cut-off number of 2 metastatic pelvic LNs, is superior at informing the prognostic outcomes of such patients than solely the qualitative presence or absence of metastatic LNs. These findings have important potential implications for therapeutic decision-making in patients with stage IIIC1 cervical cancer.

## Figures and Tables

**Table 1:** Main features of the series.

Characteristics	Patients *n*. (%)
Patients	541
Age, yrs median (range)	46 (19–85)
Histology	
Squamous	345 (63.8)
Other	196 (36.2)
Grading	
G1/G2	305 (56.3)
G3	236 (43.6)
Tumor Size	
≤2 cm	225 (41.6)
>2 cm ≤4 cm	266 (49.2)
>4 cm	50 (9.2)
LVSI *	
No	297 (54.9)
Yes	244 (45.1)
Involvement of the upper third of vagina	
No	482 (89.1)
Yes	59 (10.9)
Parametrial involvement	
No	499 (92.2)
Yes	42 (7.8)
Radicality of surgery	
Type A	52 (9.6)
Type B	226 (41.8)
Type C	263 (48.6)
Surgical approach	
Open	258 (47.7)
Laparoscopy	201 (37.2)
Robotic	82 (15.2)
Lymphadenectomy	
Pelvic	425 (78.6)
Pelvic and aortic	116 (21.4)
*n*. of pelvic lymph nodes removed, median (range)	27 (7–92)
*n*. of aortic lymph nodes removed, median (range)	11 (2–38)
Adjuvant treatment	
No	249 (46.0)
Radiotherapy	137 (25.3)
Chemotherapy	22 (4.1)
Concomitant chemoradiotherapy	133 (24.6)

* LVSI: Lymphovascular space invasion.

**Table 2:** Crosstab comparison of the distribution of patients according to the 2009 and the 2018 FIGO staging systems.

FIGO Staging Systems		Pathological FIGO Stage 2018 *n*. (%)								Total
		IB1	IB2	IB3	IIA1	IIA2	IIB	IIIC1	IIIC2	
Pathological FIGO stage 2009 *n*. (%)	IB1	209 (50.0)	148 (35.4)	0	0	0	0	**54** **(12.9)**	**7** **(1.7)**	418 (100)
	IB2	0	0	22 (73.3)	0	0	0	**6** **(20.0)**	**2** **(6.7)**	30 (100)
	IIA1	0	0	0	30 (71.4)	0	0	**11** **(26.2)**	**1** **(2.4)**	42 (100)
	IIA2	0	0	0	0	8 (88.9)	0	**1** **(11.1)**	**0**	9 (100)
	IIB	0	0	0	0	0	22 (52.4)	**17** **(40.5)**	**3** **(7.1)**	42 (100)
Total		209 (38.6)	148 (27.4)	22 (4.1)	30 (5.5)	8 (1.5)	22 (4.1)	**89** **(16.5)**	**13** **(2.4)**	541 (100)

Bold: it underline patients taken into account in further analyses.

**Table 3:** Comparison of predictive models for disease free survival based on current FIGO 2018 staging versus the revisited FIGO 2018 stage, which stratifies the N1–2 and the *n* > 2 groups within the IIIC1p stage. Bold *p* values indicate statistical significance.

Analyses of Each Variable of the Models and of the Whole Models					Current FIGO 2018		Revisited FIGO 2018	
			Univariate Analysis		Multivariate Analysis			
		Patients *n*. (%)	β	*p* Value	HR (95% CI)	*p* Value	HR (95% CI)	*p* Value
Variables of the model	**Tumor size**							
	<2 cm	223 (42.2)						
	2–4 cm	257 (48.7)	0.718		2.00 (1.20–3.33)		2.05 (1.23–3.41)	
	>4 cm	48 (9.1)	0.430	**0.019**	1.44 (0.60–3.44)	**0.028**	1.27 (0.53–3.08)	**0.018**
	**Involvement of** **the upper third** **of vagina**							
	No	472 (89.4)						
	Yes	56 (10.6)	0.404	0.179	1.40 (0.74–2.66)	0.306	1.40 (0.74–2.67)	0.302
	**Parametrial** **involvement**							
	No	489 (92.6)						
	Yes	39 (7.4)	0.328	0.377	1.12 (0.49–2.53)	0.794	1.03 (0.45–2.37)	0.949
	**Pelvic lymph** **node metastasis**							
	No	(83.1)						
	Yes	89 (16.9)	0.319	0.218	1.06 (0.60–1.89)	0.836		
	**Pelvic lymph** **node metastasis**				-	-		
	No	439 (83.1)						
	N1-2	59 (11.2)	0.164				0.60 (0.30–1.33)	
	*n*>2	30 (5.7)	1.144	**0.003**			2.78 (1.33–5.85)	**0.006**
**Global analysis of the models**	**χ2**				9.456		21.922	
	**Degrees of freedom**				5		6	
	***p* value**				0.092		**0.006**	

## Supplementary Materials

The following are available online at https://www.mdpi.com/2072-6694/12/6/1552/s1, Table S1. Distribution of pathological features of patients with stage IIIC1p according to the number of metastatic pelvic lymph nodes. Bold *p* values indicate statistical significance, Table S2. Distribution of pattern of recurrence according to the status, and the number of metastatic pelvic lymph nodes, Figure S1. (A) Overall survival in the whole series according to the FIGO 2018 staging system; (B) OS in the whole series according to the proposed FIGO 2018 staging system. Arrows highlight the DFS curve for stage IIIC1 in panel A, as well as the subgroups N1–2 and *n* > 2 in the panel B.
