# Supplementary material for: Should the Number of Metastatic Pelvic Lymph Nodes Be Integrated into the 2018 Figo Staging Classification of Early Stage Cervical Cancer?
Source: Cancers (Basel). 2020 Jun 12;12(6):1552. doi: 10.3390/cancers12061552 (PMC7352475; doi:10.3390/cancers12061552)
Supplement: Supplementary file 1 [file cancers-12-01552-s001.pdf]

cancers

MDPI

Supplementary Materials

Should the Number of Metastatic Pelvic Lymph Nodes be Integrated into the 2018 Figo Staging Classification of Early Stage Cervical Cancer?

Luigi Pedone Anchora, Vittoria Carbone, Valerio Gallotta, Francesco Fanfani, Francesco Cosentino, Luigi Carlo Turco, Camilla Fedele, Nicolò Bizzarri, Giovanni Scambia and Gabriella Ferrandina

Table S1. Distribution of pathological features of patients with stage IIIC1p according to the number of metastatic pelvic lymph nodes.

Features	Patients with Metastatic Pelvic Lymph Nodes		*p* Value
	N1-2	N>2	
**Patients**	59	30	
**Histology**			
Squamous	38 (64.4)	18 (60.0)	
Other	21 (35.6)	12 (40.0)	0.419
**Grading**			
G1/ G2	23 (39.0)	13 (43.3)	
G3	36 (61.0)	17 (56.7)	0.697
**Tumor Size**			
≤2 cm	13 (22.0)	7 (23.3)	
>2 cm ≤ 4 cm	42 (71.2)	14 (46.7)	
>4 cm	4 (6.8)	9 (30.0)	**0.026**
**LVSI**			
No	16 (27.1)	4 (13.3)	
Yes	43 (72.9)	26 (86.7)	0.148
**Involvement of the upper third of vagina**			
No	49 (83.1)	27 (90.0)	
Yes	10 (16.9)	3 (10.0)	0.380
**Parametrial Involvement**			
No	51 (86.4)	21 (70.0)	
Yes	8 (13.6)	9 (30.0)	**0.062**
**N. pelvic lymph nodes removed**			
median (range)	28.0 (10-83)	28.5 (8-92)	0.828

Table S2. Distribution of pattern of recurrence according to the status, and the number of metastatic pelvic lymph nodes

		Pattern of Recurrence(s)					
**Pelvic Lymph Node Status**	**All**	**Pelvic Central**	**Pelvic Lateral**	**Aortic**	**Distant**	**Mixed**	***p* Value**
**Negative**	69						
**N1-2**	11	35 (50.7)	8 (11.6)	-	17 (24.6)	11 (15.9)	
**N>2**	30	3 (27.3)	-	1 (9.1)	4 (36.4)	3 (27.3)	
n.a.	7	-	2 (6.7)	1 (3.3)	4 (13.3)	3 (10.0)	**0.039**

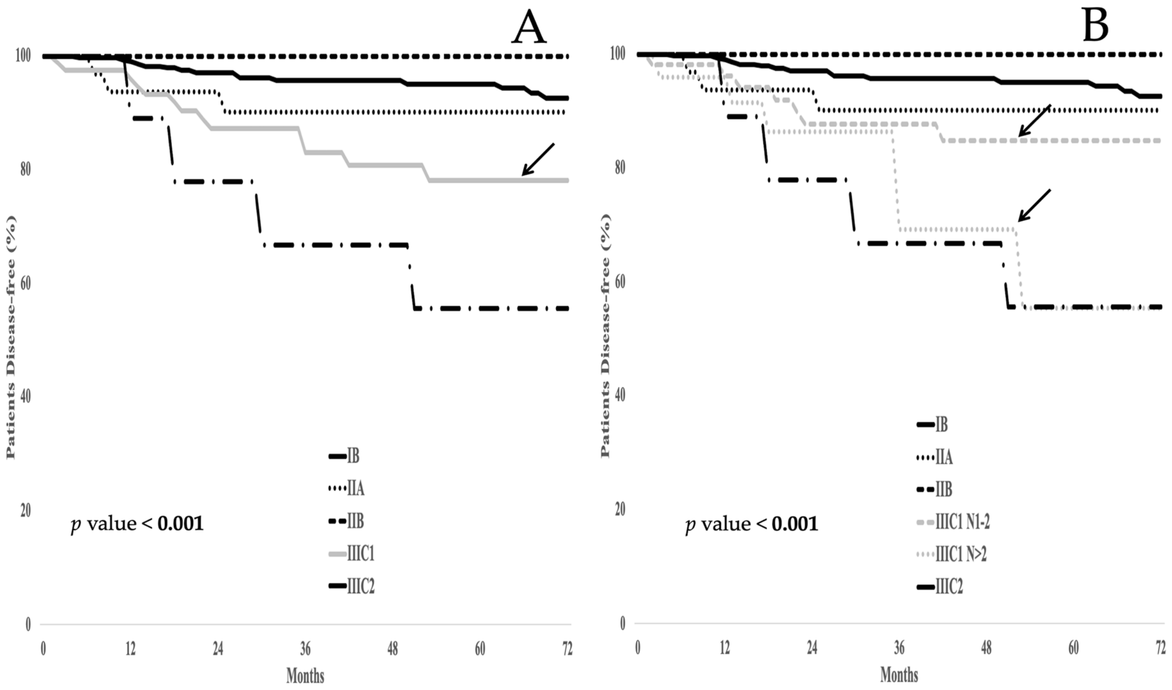

Figure 1. (**A**) Overall survival in the whole series according to the FIGO 2018 staging system; (**B**) *OS* in the whole series according to the proposed FIGO 2018 staging system. Arrows highlight the DFS curve for stage IIIC1 in panel A, as well as the subgroups N1-2 and N > 2 in the panel B.

© 2020 by the authors. Licensee MDPI, Basel, Switzerland. This article is an open access article distributed under the terms and conditions of the Creative Commons Attribution (CC BY) license (http://creativecommons.org/licenses/by/4.0/).
